# A pilot study to evaluate the combination of surgery and brachytherapy for local tumor control in young children with perianal rhabdomyosarcoma

**DOI:** 10.1016/j.ctro.2024.100862

**Published:** 2024-09-18

**Authors:** Andreas Schmidt, David Baumann, Ulf Lamprecht, Benjamin Mayer, Cristian Urla, Benjamin Bender, Jürgen Schäfer, Frank Fideler, Maximilian Niyazi, Frank Paulsen, Jörg Fuchs

**Affiliations:** a Department of Pediatric Surgery and Pediatric Urology, University Children’s Hospital, Eberhard Karls University Tuebingen, 72076 Tuebingen, Germany; b Department of Radiation Oncology, University Hospital, Eberhard Karls University Tuebingen, 72076 Tuebingen, Germany; c Department of Diagnostic and Interventional Neuroradiology, University Hospital, Eberhard Karls University Tuebingen, 72076 Tuebingen, Germany; d Department of Pediatric Radiology, University Hospital, Eberhard Karls University Tuebingen, 72076 Tuebingen, Germany; e Department of Anesthesiology and Intensive Care Medicine, University Hospital, Eberhard Karls University Tuebingen, 72076 Tuebingen, Germany

**Keywords:** Perianal rhabdomyosarcoma, Brachytherapy, Surgery, Local therapy, Young children

## Abstract

**Background:**

Perianal rhabdomyosarcoma ((P)RMS) are rare and have a poor prognosis. Data in young children are limited and local therapy is not well defined. Combined brachytherapy and surgery is routinely being used for RMS at other sites in children as it provides good oncologic outcomes and allows for organ-sparing surgery. The objective of this study was to evaluate this combination treatment for local tumor control and organ-sparing surgery in young children with (P)RMS.

**Methods:**

A retrospective review of the medical records of all children who underwent surgery and brachytherapy for (P)RMS at our institution since 2009 was conducted.

**Results:**

Surgery for (P)RMS was performed in 6 patients at a median age of 19 months (range 8–50). Embryonal RMS was diagnosed in 4 patients and alveolar RMS in 2 patients, of which 1 patient had FOXO1 fusion-positive RMS. All patients underwent postoperative high-dose rate (HDR) brachytherapy. Organ-preserving surgery was achieved in 5 of 6 patients (83 %). In 1 patient, the entire sphincter was infiltrated, making organ-preserving resection impossible. 5 of 6 patients (83 %) exhibited an event-free and overall survival at a median follow-up of 26 months (range 8–107). One patient died due to locoregional recurrence. Complications were urethral leakage in 1 patient followed by urethral stenosis and delayed wound healing and vaginal stenosis in another patient. No patient reported fecal incontinence.

**Conclusions:**

Combined treatment with surgery and HDR brachytherapy is feasible in very young children with (P)RMS and leads to a favorable oncologic outcome. Preliminary data show a good functional preservation.

## Highlights

• Pediatric perianal rhabdomyosarcoma is a rare malignancy with poor prognosis.
• Due to the rarity of the malignancy, the study includes 6 patients.
• Brachytherapy is advantageous for focusing on the tumor and sparing healthy tissue.
• It can be favourably applied with surgery for local tumor control in young children.
• The combination with surgery provides good tumor control and organ preservation.

## Introduction

Rhabdomyosarcoma (RMS) is the most common soft tissue sarcoma in children [1]. However, perianal (P)RMS is very rare, accounting for approximately 2 % of all childhood RMS cases [2]. The incidence of RMS in patients younger than 20 years of age is 4,58 per million per year [3]. In the United States, approximately 350 new cases of pediatric RMS are reported annually [4], which is equivalent to 7 cases of (P)RMS. This indicates that a reasonable number of children with (P)RMS, allowing subgroup analysis, can only be achieved through cooperative multinational studies. We conducted a pilot study comprising 6 young patients treated with combined surgery and brachytherapy (BT). To our knowledge this is the largest single center study published to date on this topic.

The prognosis for pediatric patients with (P)RMS is poor, with 5-year overall survival rates ranging from 20 % to 53 % [5], [6], [7], [8], [9], [10]. The therapeutic approach for (P)RMS typically consists of induction chemotherapy followed by local treatment, which may include surgery and/or radiotherapy. Local tumor control is essential for the success of therapy [11], and needs to be optimized to increase the currently low survival rate [5], [9]. The optimal type of local therapy is still uncertain and not clearly defined in treatment protocols. In addition to achieving an optimal oncologic outcome, the challenges of local therapy include avoiding mutilating surgery and short- and long-term sequelae of surgery and irradiation [5], [9], [12]. A significant concern is the use of external beam radiotherapy in children, which can result in acute gastrointestinal and urogenital morbidity and long-term sequelae such as functional impairment, growth retardation and reduced fertility [13], [14], [15], [16], [17]. Due to the reluctance to use radiotherapy in very young children, especially those under 1 year of age, these children tend to be undertreated and have worse outcomes compared to older children [18].

Modern BT is a radiooncologic modality that allows radiation to be effectively focused on the tumor while sparing surrounding normal tissue [19], [20], [21]. It is image guided by using MRI and CT for individualized treatment planning and uses due to its physical properties inhomogeneous dose distributions with the consequence of higher equivalent uniform dose. For bladder/prostate (BP)RMS [22], [23], [24], [25] and vaginal RMS [23], [26], [27], the combination of BT and surgery has been demonstrated to be effective for local tumor control and organ preservation, and is now well established for these tumor sites.

The purpose of our study was to evaluate the combination of BT and surgery in young children with (P)RMS in terms of oncologic and functional outcomes, organ preservation rate and incidence of complications and adverse events.

## Methods

### Patients and diagnostics

All patients with localized (P)RMS who underwent surgery and BT at our institution between 2009 and 2023 were included in the study. The diagnosis was confirmed by biopsy and subsequent histologic evaluation. FOXO1 fusion status was assessed. Lymph nodes were evaluated by MRI at diagnosis and prior to surgery. In 2 cases, a PET-MRI was performed preoperatively.

### Systemic treatment

Patients were treated according to protocol if enrolled in a clinical trial or according to the Cooperative Weichteilsarkom Studiengruppe (CWS) guidelines. Risk stratification was based on age, tumor size and histology, fusions status, and lymph node involvement. All patients received risk-adapted neoadjuvant chemotherapy. The standard regimen included ifosfamide, vincristine, and actinomycin-D. Adriamycin was added for intensification and, in the event of a poor response, carboplatine and etoposide as second-line chemotherapy.

### Local treatment

The local treatment approach was determined by a multidisciplinary team after neoadjuvant chemotherapy based on tumor size, infiltration of surrounding structures and response to chemotherapy as assessed by preoperative MRI. Inclusion criteria for the combined surgery and BT were expected R0 or marginal R1 resection and negative lymph node status prior to surgery. Sphincter involvement was not an exclusion criterion.

### Surgery

In preparation for surgery all patients underwent MRI and, in two cases, additional PET-MRI. The aim of surgery was to achieve a R0 or marginal R1 resection. In the event of involvement of the sphincter, this tumor area was also resected and the sphincter was reconstructed. A maximum of one third of the sphincter was permitted to be infiltrated by the tumor in order to perform organ-preserving surgery.

### Brachytherapy

**Fig. 1:**
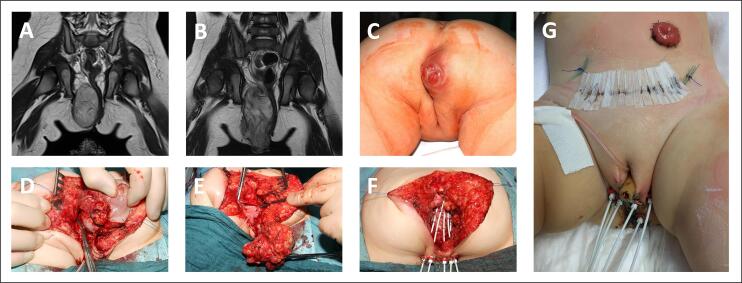
Radical resection combined with brachytherapy in a 14-month-old girl (patient #3) with embryonal, perianal rhabdomyosarcoma. A) Initial MRI. B) Preoperative MRI. C) Tumor localization. D) Situs with tumor and opened dorsal rectal wall (probe in rectum). E) Situs with tumor and opened dorsal vaginal wall. F) Situs after tumor resection and after placement of brachytherapy tubes. G) Position of brachytherapy tubes, urinary catheter and probe in the vagina, ovaries transpositioned to the anterior abdominal wall.

**Fig. 2:**
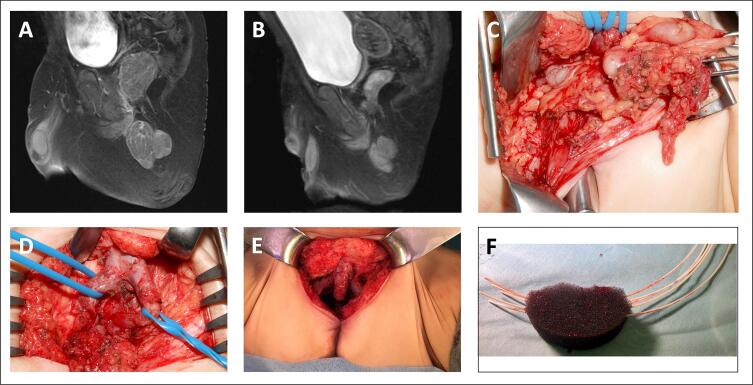
Organ-preserving resection combined with brachytherapy in a 20-month-old boy (patient #4) with embryonal, perianal rhabdomyosarcoma. A) Initial MRI. B) Preoperative MRI. C) Situs before tumor resection. D) Situs after tumor resection with view from perineal. Corpora cavernosa marked by blue loops. E) Situs after tumor resection. F) Sponge with brachytherapy tubes before placement in the wound cavity. (For interpretation of the references to colour in this figure legend, the reader is referred to the web version of this article.)

**Fig. 3:**
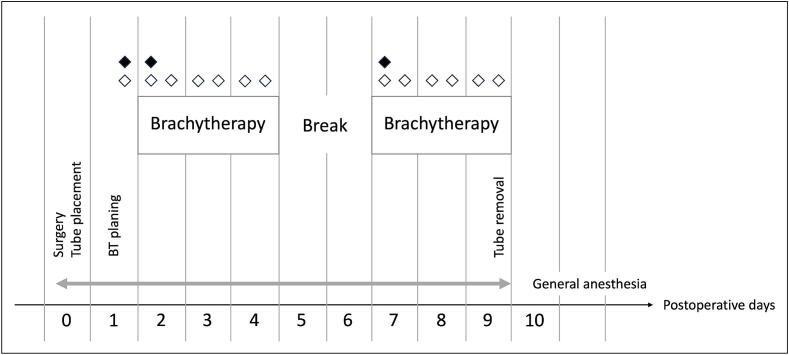
Schedule of combination therapy for children with perianal rhabdomyosarcoma. BT is performed over 6 days with a break of 2 days after half of the time. General anesthesia (gray arrow) lasts from the time of surgery until the BT tubes are removed at the end of radiotherapy. Control of the position of the tubes by MRI on the day of surgery. Planning of BT on the first postoperative day. Verification of tube placement before the start of radiotherapy and then at regular intervals (◊ control by external tube length, ♦ fluoroscopic control).

**Fig. 4:**
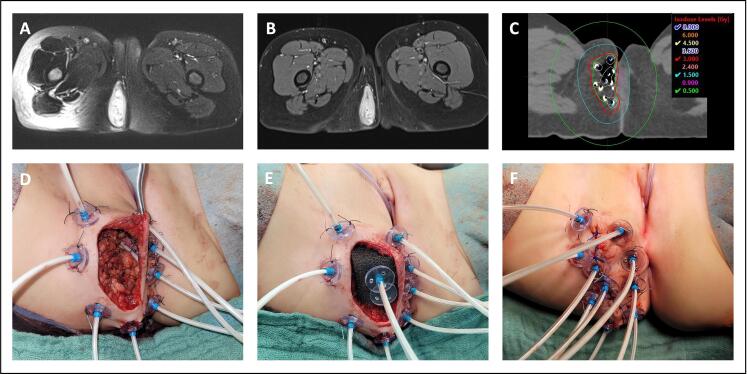
Organ-preserving resection combined with brachytherapy in a 50-month-old girl (patient #6) with embryonal, perianal rhabdomyosarcoma. A) Initial MRI. B) Preoperative MRI. C) Radiation treatment planning showing target volume (inner green line), organs at risk (vagina (purple line) and rectum (brown line)) and isodose lines per fraction. D) Situs after tumor resection and placement of brachytherapy tubes. E) Situs after additional placement of a sponge with brachytherapy tubes into the wound cavity. F) Position of the tubes after closure of the skin over the sponge.

Following tumor resection, BT tubes were placed at the margins of the former tumor area (Fig. 1F). The aimed distance between two tubes was ≤1 cm in order to achieve homogeneous coverage of the target volume while maximizing protection of healthy surrounding tissue. A sponge, originally developed for vacuum-assisted wound closure, was used in selected cases to facilitate the placement of additional BT tubes and to hold the tubes in place (Fig. 2F, Fig. 4E). The sponge was positioned within the wound cavity, with the tubes inserted and secured with sutures. The skin was then closed over the sponge (Fig. 4F). Once the correct position of the BT tubes had been verified by postoperative CT and MRI and from year 2021 onwards by intraoperative MRI (iMRI) [28], the irradiation parameters were calculated and the irradiation was initiated on postoperative day 2 (Fig. 3). MRI is additionally used because of its superior tissue resolution compared to CT, a better visualization of the organs at risk and the position of the BT tubes in relation to the anatomical structures, and a more precise definition of the target volume. The BT protocol is adapted from the protocol for percutaneous radiation therapy of the *CWS*. Irradiation was delivered as high-dose rate (HDR)-BT with a 192Ir source at a total dose of 36 Gy in 12 fractions, twice daily. Following three days of irradiation, there was a two-day break in irradiation at the weekend for tissue recovery. Patients are under general anesthesia including neuromuscular blockade, initiated for surgery, from the time the BT tubes are placed until they are removed to prevent the tubes from shifting and therefore unplanned dose distribution. The position of the tubes is checked regularly (Fig. 3). The procedure is identical to that described for (BP)RMS [22], [23].

### Follow-up examinations

Follow-up examinations were conducted in accordance with the current CWS protocol. MRI of the former tumor region and the pelvis was obtained every 3 to 4 months during the first year after local therapy and every 6 months thereafter. Staging examinations of the lungs were conducted every six months and of other sites on a risk-adjusted basis. During hospitalization for MRI, the children underwent a comprehensive clinical examination, which included an assessment of wounds and scars, as well as of the anus and the perianal region.

### Statistical analysis

Data were collected from patient records and analyzed retrospectively. We considered patient and tumor data, event-free and overall survival, surgical and radiation procedures, and the occurrence of complications and adverse events. Event free is defined as the absence of tumor progression or relapse. Descriptive statistics were used for analysis. Data are presented as median and range. The study was approved by the Ethics Committee of the University of Tuebingen, Germany (No. 974/2021BO2).

## Results

### Patients

6 children (4 males, 2 females) were included in the study. The median age at the time of surgery was 19 months (range 8–50). Patient #1 exhibited suspicious inguinal and iliac lymph nodes at the time of diagnosis. However, preoperative imaging after neoadjuvant chemotherapy showed no evidence of lymph node metastasis. Histopathology revealed that 4 patients had embryonal RMS, while 1 had fusion-positive (FOXO1-Pax 3-gene) alveolar RMS and 1 had fusion-negative alveolar RMS. 4 patients were classified as high risk, 1 as standard risk, and the fusion-positive alveolar RMS patient with the initially suspicious lymph nodes was classified as very high risk. Neoadjuvant chemotherapy failed to shrink the tumor in patient #3 despite a change in neoadjuvant chemotherapy (Table 1)

**Table 1:** Patient characteristics, tumor characteristics and outcome parameter.

Patient #	Age at surgery [months]	Gender	Histology; FOXO1 fusion status	Tumor size [cm] (at diagnosis/preoperative)	Lymph node (at diagnosis/ preoperative)	Resection status	Surgery	Sphincter invasion	Follow up [months]	Outcome
1	8	M	alveolar; positive	5.0/1.2	suspicious/negative	R1	organ sparing	partial	15	dead (loco-regional recurrence)
2	18	M	embryonal; negative	6.3/3.8	negative/negative	R1	organ sparing	partial	107	NED
3	14	F	embryonal; negative	5.3/7.0	negative/negative	R0	mutilating	total	80	NED
4	20	M	embryonal; negative	4.7/4.0	negative/negative	R1	organ sparing	no	37	NED
5	22	M	alveolar; negative	4.8/2.8	negative/negative	R0	organ sparing	partial	12	NED
6	50	F	embryonal; negative	5.6/3.7	negative/negative	R0	organ sparing	partial	8	NED

NED no evidence of disease.

### Surgery

Organ-sparing resection was achieved in 5 patients (83 %). In 4 of the 5 cases, parts of the external anal sphincter were resected and the sphincter muscle was reconstructed. In patient #4, the tumor did not involve the sphincter but extended to the level of the bladder neck and the pelvic floor and was resected perineally and abdominally. The remaining 4 patients underwent perineal resection only. R0 resection was achieved in 3 patients and R1 resection in 3 patients (Table 1).

Patient # 3 exhibited a complete infiltration of the sphincter muscle, making preservation of the organ impossible. Therefore, partial pelvic exenteration with abdominoperineal rectal resection and creation of a terminal anus praeter and a partial resection of the posterior vaginal wall with subsequent reconstruction were performed.

### HDR- Brachytherapy

All patients received BT after tumor resection. A median of 7 tubes (5–10) were placed for BT. The number varied depending on the size of the irradiation field. In 3 cases, a sponge was inserted into the wound cavity to facilitate tube placement and to avoid tube distortion. Dosimetric parameters are listed in Table 2.

**Table 2:** Dosimetric parameters of the treated patients per fraction.

Pat #	CTV	Rectum	Rectum	Rectum	Urethra	Urethra	Growth Plate	Growth Plate
	D90%	D1cc	D0.1 cc	D0.01 cc	D0.1 cc	D0.01 cc	D0.1 cc	D0.01 cc
1	3.6	3.0	4.1	5.2	2.6	3.1	1.5	1.9
2	3.7	2.0	2.7	3.1	3.6	4.2	0.6	0.7
3	3.0	−	−	−	1.9	2.1	2.1	2.4
4	3.3	2.9	3.7	4.3	2.7	3.6	2.5	2.9
5	3.2	2.2	2.7	2.9	2.8	4.0	0.7	3.0
6	2.8	1.9	2.5	2.7	0.9	1.1	1.0	1.4
Constr.	3.3	2.0	3.0	3.6	4.0	4.6	1.5	1.9

Patient 3 had a rectal exstirpation due to tumor extension. CTV: clincal target volume; Growth Plates: growth plates (highest values of both sides); D90 %: dose in 90 % of the target volume; Dxcc: highest dose in x ml of the organ; Constr.: constraints derived from former experience in bladder-prostate rhabdomyosarcoma.

In patient #3, who had undergone a rectal resection, it was also decided that BT would be a more appropriate treatment option than external beam irradiation due to the patient́s young age of 14 months. Ovarian transposition to the anterior abdominal wall was performed for the duration of radiation therapy and the irradiation dose was delivered as in the other patients.

### Complications

One patient (#2) presented with urethral stenosis distal to the pars prostatica after postoperative urinary leakage. The stenosis was resected using a laser 21 months after local therapy. Voiding was normal and no evidence of re-stenosis was observed in the micturition cysto-urethrography performed several months later. He occasionally wetted himself during the day, but this improved with consistent, regular voiding. There was no bed-wetting at night at the last follow-up examination (107 months).

The patient who underwent radical tumor resection (#3) exhibited postoperative delayed wound healing dorsal to the vagina/perineum, which resolved with regular alginate dressing changes. In addition, vaginal stenosis was noted at routine follow-up. Corrective surgery is planned for this patient at approximately 10 years of age.

### Follow-up

A median follow-up of 26 months (range 8–107) was observed, with 5 of 6 patients (83 %) still alive at the time of analysis. The patient with fusion-positive alveolar RMS (#1) developed a local and nodal relapse 15 months after local therapy and subsequently died 6 months later (Table 1).

4 of the 5 patients with organ preservation are still alive. Three were wearing a diaper at the last follow-up visit. Fecal incontinence was not reported in any of the patients.

## Discussion

The organ- and function-preserving combined approach with limited surgery and radiotherapy might use modern percutaneous irradiation techniques as photon- or proton-IMRT/IGRT or BT. The decision on using protons or photons vs BT in this rare entity depends individually on the desired target volume (local tumour extension, inclusion of lymph node areas), the dosimetric inclusion of organs at risk and on the experiences of the treating interdisciplinary teams. Percutaneous applied radiotherapy might irradiate larger volumes with relevant doses than our interstitial treatment even with modern techniques. The interdisciplinary inspection and the influence on the anatomy of tumor and organ at risk region within the surgical procedure leads to an optimization of local dose distribution. Perianal tumors allow a localized treatment with the need of localized dose delivery to critical structures (anal sphincter).

We evaluated the combination of surgery and BT in 6 young patients with (P)RMS treated at our center. (P)RMS are typically localized close to the body surface but may extend widely, contact or infiltrate adjacent tissues, and often metastasize to lymph nodes. In this pilot study, preoperative negative lymph node status and R0 or marginal R1 resection were required for combination therapy. Infiltration of the sphincter did not preclude patients from combination therapy; however, two-thirds of the sphincter had to be free of tumor for successful sphincter reconstruction. The frequent proximity of (P)RMS to the pelvic bones, the corpora cavernosa, the urethra and the vagina presents a significant challenge in the resection of these tumors. In most cases, only a marginal R0 or R1 resection is feasible. Therefore, additional radiotherapy is an essential component of the treatment. External beam radiation is known to have significant side effects, especially in very young children. For this reason, BT is also particularly suitable for this tumor site.

One of the patients exhibited local and nodal relapse 15 months after local therapy and subsequently died six months later. The patient was classified as high risk due to the presence of fusion-positive alveolar RMS. Furthermore, the patient exhibited suspicious inguinal and iliac lymph nodes at the time of diagnosis. It is evident that BT does not encompass these lymph node regions. However, the lymph nodes were not identified as suspicious prior to surgery, indicating a favorable response to neoadjuvant chemotherapy. This was the first patient in the series. The remaining patients did not exhibit a fusion-positive status or any positive lymph nodes at diagnosis. Based on this single experience, BT as the sole radiotherapeutic modality for patients with suspicious lymph nodes at diagnosis should be considered critically, even if the lymph nodes are responding to neoadjuvant chemotherapy. The recurrence was also observed in the former tumor region, indicating that the aggressiveness of the tumor may have contributed to the recurrence and that the event may not be solely attributed to lymph node activity.

In general, regional lymph node management is a critical issue in (P)RMS [6], [29]. Of all RMS localizations, (P)RMS is known to metastasize most frequently to locoregional lymph nodes [6], [9], [30], [31]. The combined results of three multinational studies of (P)RMS show that lymph node involvement occurs in 46 % of the patients. The incidence of lymph node involvement was lower in children younger than 10 years. The highest rate of lymph node metastasis was observed in patients with large alveolar tumors (78 %). Lymph node relapse occurred in 17 % of patients, all of whom were female and had alveolar tumors > 5 cm in size [9]. The recommendations for lymph node management vary. Some authors advocate for surgical evaluation of the regional lymph nodes in all patients [6], [9], while others propose prophylactic irradiation of the ilioinguinal lymph nodes in all patients older than 10 years [8]. Some authors recommend a PET scan [32], [33], which was performed in 2 of our patients. None of our patients met the criteria of age ≥10 years for prophylactic radiotherapy as recommended by Casey [6]. We performed MRI, which provides better tissue resolution than a CT, both for diagnosis and for planning surgical resection.

Organ preservation was achieved in 5 of 6 children. In one child, the sphincter was completely infiltrated by the tumor, making organ preservation impossible. Consequently, a rectal extirpation was performed. However, the patient also received BT due to her young age and the aforementioned advantages compared to external beam radiotherapy.

Due to the more superficial location of the (P)RMS tumor compared to the (BP) RMS and the wound cavity that inevitably results from tumor resection, placement of the BT tubes was particularly challenging. We have developed a method in which a sponge is placed in the wound cavity to hold the BT probes in position at the edge of the former tumor area, and in which additionally BT probes can be inserted for irradiation.

General anesthesia and neuromuscular blockade were used to prevent patient movement and tube dislocation. In pulsed-dose rate BT in children, general anesthesia was not applied and tube dislocation was reported [34]. Uncertain patient cooperation and no tolerance for tube dislocation are reasons for general anesthesia [35]. A standardized anesthesia and sedation protocol was implemented to prevent withdrawal and delirium and to achieve early extubation and discharge from the pediatric intensive care unit [35].

There have been few reports of BT specifically for perianal RMS in children, although an initial report of a larger cohort of children treated with HDR BT was published as early as 1995 [36]. These are generally limited to individual case reports and enumeration of BTs performed in multicenter trials, often without detailed individual patient and tumor characteristics [5], [9], [23], [37], [38]. Recently, Rogers in a report on SIOP MMT95, Italian RMS 96 and EpSSG RMS 2005 mentioned 5 patients treated with BT in RMS 2005 and 1 patient in MMT 95 [9]. 3 of these patients were treated with a combination of external beam RT (EBRT) and a BT boost. All 6 tumors exhibited alveolar histology, 3 of 5 were fusion positive. 5 patients survived. One patient with a 6 cm, fusion positive tumor and N1 died after receiving BT and EBRT due to local relapse outside the irradiation field. General recommendations for BT in RMS in children based on expert opinion and experience have recently been published [39]. These recommendations have also been incorporated into the radiotherapy guidelines of the ongoing FaR-RMS study [40].

The median age of the children in our study was 19 months (range 8–50 months). Regarding younger age as a predictor of oncologic outcome, the results are mixed; European and US studies have reported a worse outcome in children with RMS younger than 1 year compared to older children [18], [41], [42]. This is due to concerns about the administration of aggressive therapy, especially radiotherapy, in these infants. Indeed, an analysis of the Children’s Oncology Group trials ARST0331 and ARST0531 in children ≤24 months of age showed that the local recurrence rate was increased in children not treated according to protocol, mainly due to a reduction in radiotherapy [18]. In the context of the reluctant use of radiotherapy, surgery is becoming increasingly important in these children [43]. However, the results of the EpSSG RMS 2005 study showed no worse outcome in children <12 months of age compared to children 12–36 months of age. The 5-year overall survival was significantly better (88.4 % vs. 78.0 %; p = 0.0204) and the 5-year event-free survival was comparable (72.5 % vs. 66.1 %; p = 0.2663) [37]. In addition to possible favorable patient characteristics and improved diagnostic and therapeutic modalities, the authors attribute these good results to an increased use of appropriate local therapy. In conclusion, all these studies suggest that the appreciation of a therapeutic modality and its appropriate use contribute to a favorable oncologic outcome.

For this pilot study, the interpretation of the study results is inherently limited by the small sample size and a brief follow-up period. Consequently, the results must be interpreted with these limitations in mind. Due to the small number of cases, the analysis was only descriptive. The retrospective nature of the study may increase the impact of confounding factors.

The follow-up period of our study is too short to cover late sequelae. After external beam radiotherapy for (P)RMS in patients enrolled in various multicenter studies, 3 of 5 patients who responded to a questionnaire sent to all survivors reported fecal incontinence, no bowel motility or voiding problems [5]. In general, patients with RMS are at increased risk of developing sequelae [44], depending on the radiotherapeutic modality [45].

The patients in our study do not represent the full spectrum of children with (P)RMS. Age ≥ 10 years, tumor size ≥ 5 cm, lymph node involvement, alveolar histologic subtype and fusion-positive status have been described as prognostically unfavorable parameters [5], [6], [8], [9]. However, not all studies demonstrated an association between histologic subtype, fusion status, or lymph node involvement and outcome [6], [8], [9]. This may be attributed to lack of statistical power [9]. In our study, no patient was older than 5 years. As more data become available, it may be possible to define a broader use of combined BT and surgery in (P)RMS. A combination of BT and EBT as reported by Rogers [9] may also permit the use of BT in the case of lymph node involvement.

## Conclusion

This pilot study shows that the combination of surgery and BT in young children with (P)RMS is feasible and provides good oncologic and functional outcomes in selected patients. The results of this pilot study need to be confirmed in a larger study and with a longer follow up. An extension to the patient profile not covered in our study will show whether this therapeutic approach is more broadly applicable to the treatment of (P)RMS. Due to the rarity of the disease, only multinational cooperative trials can provide these data.

## Funding

This research received no external funding.

## CRediT authorship contribution statement

**Andreas Schmidt:** Conceptualization, Visualization, Writing – original draft. **David Baumann:** Investigation, Writing – review & editing. **Ulf Lamprecht:** Investigation, Writing – review & editing. **Benjamin Mayer:** Resources, Writing – review & editing. **Cristian Urla:** Resources, Writing – review & editing. **Benjamin Bender:** Investigation, Writing – review & editing. **Jürgen Schäfer:** Investigation, Writing – review & editing. **Frank Fideler:** Investigation, Writing – review & editing. **Maximilian Niyazi:** Investigation, Writing – review & editing. **Frank Paulsen:** Conceptualization, Supervision, Writing – review & editing. **Jörg Fuchs:** Conceptualization, Supervision, Writing – review & editing.

## Declaration of competing interest

The authors declare that they have no known competing financial interests or personal relationships that could have appeared to influence the work reported in this paper.

## Data Availability

All data generated or analysed during this study are included in the published article.
